# More connections, higher polarization: The role of weak ties and status-driven dynamics

**DOI:** 10.1073/pnas.2614422123

**Published:** 2026-06-29

**Authors:** Piotr J. Górski, Giacomo Vaccario, Janusz A. Hołyst, Michael W. Macy

**Affiliations:** ^a^ Faculty of Physics, Warsaw University of Technology, Warsaw 00-662, Poland; ^b^ Chair of Ecosystem Management, Department of Environmental Systems Science, ETH Zürich, Zürich 8092, Switzerland; ^c^ Department of Sociology, Cornell University, Ithaca, NY 14853; ^d^ Department of Information Science, Cornell University, Ithaca, NY 14853

Social polarization is central to contemporary scientific and public discourse. Thurner et al. (1) attribute rising polarization to increased social interaction, operationalized as growth in close friendships, within a framework combining homophily and structural balance. However, as noted in ref. 2, the evidence for such an increase cannot be completely justified, and an opposing narrative of a “friendship recession” has long been discussed (3). Here, we complement (1) by showing that increasing connectivity can indeed promote polarization, but through a different mechanism: the expansion of lower-intensity (weak) ties, which amplifies status-driven dynamics.

Technological and societal changes have enabled long-distance interactions, previously weak or inexistent, to become more frequent and meaningful (4, 5). Still, such ties often remain distinct from close friendships. Indeed, empirical evidence points to growth in weak ties and acquaintances rather than emotionally close relationships (6).

**Fig. 1.**
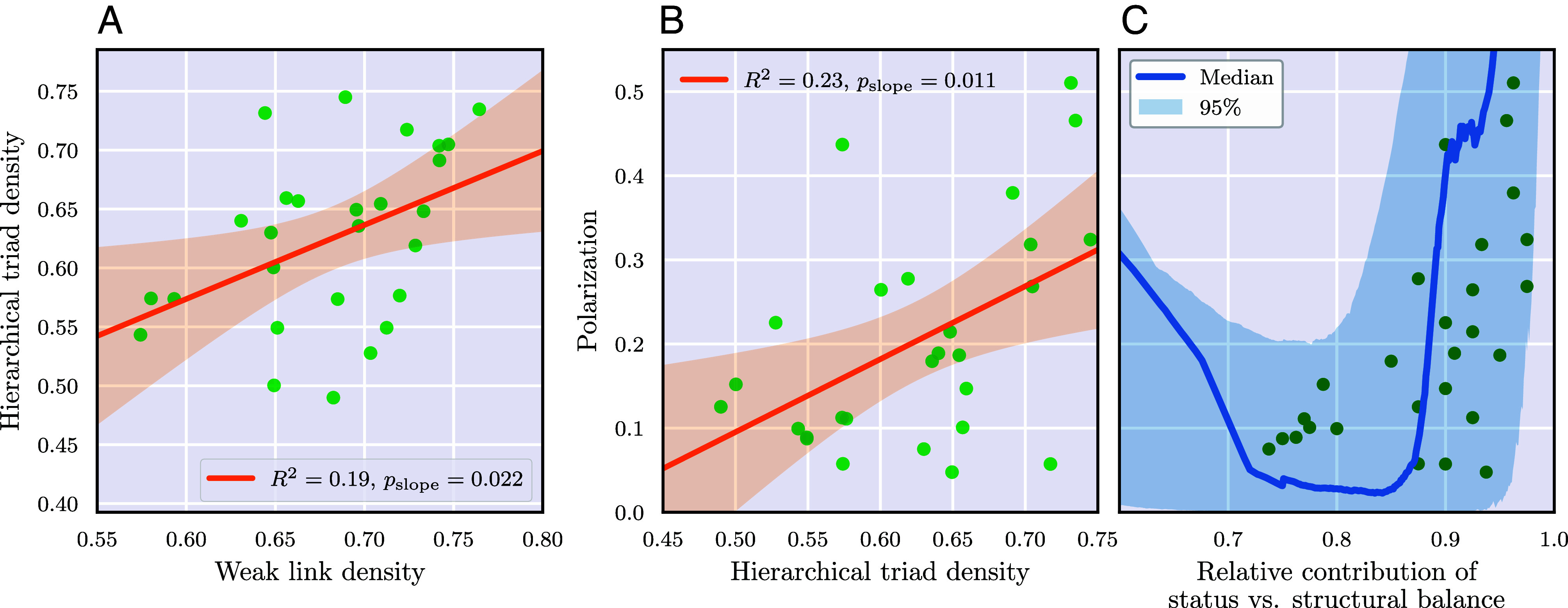
A pathway to polarization through weak ties and status-driven dynamics. Panels (*A* and *B*) explore empirical data from the Spanish High School dataset (SHSD), comprising signed student networks with information on tie strength (7). Lines show OLS fits. In all panels, points represent distinct school networks, and shaded areas denote 95% CIs. Panel (*C*) presents polarization as a function of relative contribution of status versus structural balance, as obtained from the agent-based model calibrated to SHSD. The blue curve represents the median analytical prediction based on parameter sampling that preserves empirical cross-correlations (8).

The increasing prevalence of weak ties relative to strong ties raises important questions about their role in social networks. In multiple-class signed school networks (7), a higher proportion of weak ties is positively correlated with a higher hierarchical triad density (Fig. 1*A*). Hierarchical triads are motifs consisting of three-edge patterns not violating status theory (8). Moreover, networks with more hierarchical triads tend to be more polarized (Fig. 1*B*). We quantify polarization as the density of triadic patterns containing two or three negative links (9), consistent with the idea that polarization reflects the division of a system into antagonistic groups, whereas homophily-based models emphasize division based on disagreement in node attributes.

The interplay between weak ties, status, structural balance, and polarization can be understood by noting that sign formation depends on tie strength. Structural balance operates by resolving cognitive dissonance in inconsistent triads. When ties are weak, such inconsistencies are less salient, reducing the pressure to restore balance (10). Consequently, relations are increasingly shaped by positional considerations (status). In line with homophily, strong ties tend to connect similar individuals. Thus, weak ties are more likely to connect dissimilar ones, increasing the role of status.

Consistent with this interpretation, a calibrated agent-based model (8) shows that the relative importance of status over structural balance increases with weak-link density. Model analysis further reveals that a greater role of status amplifies polarization (Fig. 1*C*). This result holds when status dominates, i.e., when status considerations are applied more than twice as frequently as structural balance, corresponding to parameter values above ∼0.7. Empirically inferred values of status importance for the analyzed school networks lie within this regime.

Homophily connects relation formation to attribute similarity, creating a feedback between disagreement and antagonistic ties. While this mechanism can drive polarization (1), our results suggest an alternative pathway: Technological change promotes weak ties, which elevate the role of status-based mechanisms and increase polarization.

In summary, not all social interactions are equivalent. Tie strength determines which social mechanisms dominate network evolution. Accounting for this interplay between interaction strength and multiple mechanisms, including homophily, structural balance and status, is therefore essential for understanding the drivers of polarization.
